# Effects of dietary copper intake on blood lipids in women of childbearing age and the potential role of gut microbiota

**DOI:** 10.3389/fnut.2024.1368730

**Published:** 2024-03-05

**Authors:** Mingming Luo, Linmei Guo, Chunmei Wu, Ming Hao, Junwang Gu, Xuhuan Li, Qi Wang

**Affiliations:** ^1^Jiangxi Cancer Hospital, The Second Affiliated Hospital of Nanchang Medical College, Jiangxi Cancer Institute, Nanchang, China; ^2^Department of Health Statistics, School of Public Health and Health Management, Gannan Medical University, Ganzhou, China; ^3^The Fourth Affiliated Hospital of Nanchang University, Jiangxi Medical College, Nanchang University, Nanchang, China

**Keywords:** copper, blood lipids, dietary Cu intake, women of childbearing age, gut microbiota

## Abstract

**Background:**

Copper (Cu) is a vital trace element involved in numerous physiological processes, including glycolysis and lipid metabolism. Imbalances in Cu homeostasis can contribute to various diseases. However, current research on the impact of Cu on lipid metabolism has yielded inconsistent findings. Moreover, studies investigating the effects of dietary Cu intake on blood lipids among women of childbearing age are rare. Understanding of this relationship could enhance lipid management, given that most women obtain Cu through their diet. Additionally, the gut microbiota may play a role in this process. This study aims to investigate the effects of dietary Cu intake on blood lipids in women of childbearing age and to analyze the role of gut microbiota in this process.

**Methods:**

This study utilized data from the National Health and Nutrition Examination Survey (NHANES) to conduct a preliminary analysis of the correlation between dietary Cu levels and blood lipid indicators in women of childbearing age. Subsequently, an on-site research was conducted to further investigate this relationship, followed by animal experiments to verify the effect of different Cu doses on blood lipid levels. Multiple linear regression models, ANOVA, XGBOOST were employed to analyze the impact of Cu on blood lipids and the role of intestinal microbiota in this process.

**Results:**

In the population study, the NHANES results were consistent with on-site findings. The TG, and TC levels in women with childbearing were increased with higher dietary Cu intake. Animal experiments have shown that as Cu intake increases, TC levels increase. Furthermore, when the Cu intake reached 8 mg/day (the recommended dietary Cu intake limit of China, RDI), the TG levels in the research animals decrease, alongside a reduction in the abundance of *Weissella cibaria* (probiotics related to lipid metabolism), and the levels of LPS and IL-6 increase.

**Conclusion:**

The blood lipid levels of women of childbearing age increase with higher dietary Cu intake. RDI of 8 mg/day for women of childbearing age in China may need to be appropriately reduced. Regulating the gut microbiota, especially by increasing the abundance of *Weissella cibaria* may be an effective intervention for blood lipids.

## 1 Introduction

Copper (Cu) is a crucial cofactor for many enzymes involved in various physiological processes, including energy metabolism, oxygen transport, cell metabolism, peptide hormone maturation, blood coagulation, signal transduction, and various other processes (1). Imbalances in Cu homeostasis (either deficiency or excess) can contribute to the onset and progression of various diseases, including neurological disorders, tumor advancement, and heightened tumor invasiveness (2–4). Furthermore, disorders in Cu homeostasis are also associated with alterations in glycolysis, insulin resistance, and lipid metabolism (5–7).

However, current research on the impact of Cu on lipid metabolism has yielded inconsistent findings. A study on the influence of Cu on the physiological function of adipocytes suggested that severe Cu deficiency in the body can lead to triglyceride accumulation (8). Moreover, study conducted by Liu et al. (9) also indicated that supplementing Cu can reduce lipid levels. Conversely, the findings of other researchers propose that an increase in Cu content in the body is associated with obesity and elevated blood lipids (10, 11). The existing research primarily focuses on *in vitro* studies, *in vivo* studies of single animal models, or investigations into the internal exposure levels of Cu in the general population. Few studies have directly explored the influence of dietary Cu intake on blood lipids, especially in women of childbearing age (18–45 years old). Abnormal blood lipid levels in women of childbearing age not only elevate their own risk of chronic diseases but also increase the risk of chronic diseases in their offspring through intergenerational transmission. Simultaneously, it can also lead to a decrease in the age at which chronic diseases occur in their offspring (12). Therefore, it is essential to comprehend the impact of Cu on blood lipids in women of childbearing age to provide more precise guidance for lipid management.

Compared to occupationally exposed individuals, women of childbearing age mainly rely on dietary intake for Cu. Dietary intake of Cu is absorbed through the intestines and enters the human body to exert its effect (1). Hundreds of millions of microorganisms inhabit the intestine (13), and exposure to metal compounds has been demonstrated to alter the diversity and composition of the gut microbiota (14, 15). Existing evidence has established that the gut microbiota can directly or indirectly participate in numerous metabolic pathways of the body, thereby influencing host function (16). Whether Cu intake affects gut microbiota and subsequently impacts overall health? This also requires further in-depth research. Further research is needed.

Building on this premise, this study aims to investigate the influence of dietary Cu intake on the blood lipids of women of childbearing age through the National Health and Nutrition Examination Survey (NHANES) database, on-site epidemiological data, and animal experiments. At the same time, we also seek to comprehend the potential impact of Cu on gut microbiota and analyze how Cu may affect blood lipids through the gut microbiota pathway.

## 2 Methods

### 2.1 Study population

#### 2.1.1 The NHANES population

A preliminary analysis of the association between dietary Cu levels and blood lipids was conducted using data from the NHANES spanning from 2003 to 2020. The NHANES data can be accessed at https://www.cdc.gov/nchs/nhanes/. After excluding data that did not meet the specified criteria (such as males, patients with tumors, missing blood lipid or dietary data, and those outside the target age range), eligible women of childbearing age were selected for the analysis.

#### 2.1.2 The on-site survey population

To further validate the research findings from NHANES, we conducted a dietary survey on women of childbearing age who underwent physical examinations at a tertiary hospital from July to August 2022. Basic information and blood lipid data of the study subjects were collected. Dietary data were referred to the “Chinese Food Composition Table” (17) to calculate dietary Cu intake levels. Patients with liver and kidney diseases, tumors, and those unwilling to cooperate were excluded from the study. After excluding individuals with incomplete dietary data and missing blood lipid data, a total of 540 eligible research subjects were included. This study has been approved by the Ethics Committee of Gannan Medical University, and informed consent has been obtained from all research subjects.

### 2.2 Animal experiments

27 female Wistar rats (21 days old) were bred and maintained in a specific pathogen-free environment with a temperature of 22 ± 2°C, humidity of 60 ± 10%, and 12 h of dark circulation. After 1 week of adaptive feeding, they were evenly divided into a control group, a low-dose group, and a high-dose group (0, 1, and 4 mg/kg • BW Cu^2+^ solution, respectively) based on similar body weight, with nine animals in each group. The Cu-containing solution was intragastrically administrated to rats at each group for 6 weeks. During the intervention period, rats were weighed, and their food intake was recorded at a fixed time every day. After the intervention, the rats were anesthetized and euthanized after fasting for 12 h. Fecal samples were promptly collected from all rats for the detection of gut microbiota levels. Prior to euthanasia, blood samples were obtained from all rats and serum was isolated by incubating the blood at room temperature for 2 h, followed by centrifugation at 2,000 g/min for 10 min at 4°C. The collected serum was taken to measure blood lipids, Cu, LPS, and IL-6 levels. The liver and abdominal fat were weighed, and organ coefficients were calculated.

#### 2.2.1 Dose selection basis

The Cu exposure level of the population is about 1.2 mg/day, and the average daily dietary Cu intake is calculated to be about 0.024 mg/kg based on an average body weight of about 50 kg. By selecting 25–50 times the equivalent dose (18), the low dose level of Cu exposure in rats is determined to be 1 mg/kg. Based on the recommended daily upper limit of Cu intake of 8 mg/day, the high dose level of Cu exposure in rats is determined to be 4 mg/kg.

Fecal samples were collected into sterile tubes, immediately frozen in liquid nitrogen, and stored at −80°C until analysis. Gut microbiotas from each rat were detected using the 16S rRNA gene sequencing method. LPS and IL-6 levels were analyzed using an enzyme-linked immunosorbent assay (ELISA) kit, the blood lipid levels were detected according to the kits. Cu levels in the serum were measured using ICP-MS. All animal procedures were conducted in compliance with the Guide for the Care and Use of Experimental Animals and were approved by the Animal Ethics Committee of Gannan Medical University (Approval number: 2022329).

### 2.3 Statistical analysis

Quantitative data are presented as mean ± standard deviation, while qualitative data is expressed as frequency (%). Simple linear regression was used to explore the correlation between various factors and blood lipid indicators. Multiple linear regression was used to construct model I-V to adjust for covariates and investigate the relationship between Cu and blood lipid indicators. For animal research, food intake and body weight were analyzed by two-way repeated measure ANOVA, while other indicators were compared using one-way ANOVA. A ternary phase diagram was employed to depict the distribution of gut microbiota abundance in the top 10 of the three groups at each level. The intersection relationship of microbial operational taxonomic units (OTUs) among groups was represented using a Venn diagram. XGBOOST modeling was used for the screening of important gut microbiota, and species abundance was compared using the Wilcoxon rank sum test. All statistical analyses were conducted in R 4.3.1, with a significance level set at *p* < 0.05.

## 3 Results

### 3.1 Correlation between dietary Cu intake and blood lipid indicators in the NHANES study

A total of 5,326 participants in NHANES were tested for LDL and met the inclusion criteria. Among women of childbearing age, the LDL level was found to be 2.789 ± 0.848 mmol/L. Univariate analysis revealed that age, fast glucose level, BMI, education level, race, country, history of hypertension, smoking, vigorous physical activity (PA), marital status, and alcohol consumption had statistically significant effects on LDL levels. 1,072 qualified female research subjects were tested for HDL. Among women of childbearing age, the HDL level was found to be 1.538 ± 0.430 mmol/L. Univariate analysis revealed that age, fast glucose level, BMI, dietary Cu levels, dietary fiber, dietary zinc, education, race, country, history of hypertension, smoking, moderate physical activity, marital status, and alcohol consumption had a statistically significant impact on HDL levels. A total of 11,271 eligible female research subjects were tested for TG, revealing that the TG level of women of childbearing age is 1.385 ± 1.196 mmol/L. Apart from smoking status and dietary cholesterol, univariate analysis indicated that dietary Cu levels and other covariates had a statistically significant impact on TG. Additionally, a total of 11,279 eligible female subjects were tested for TC, showing that the TC level of women of childbearing age is 4.89 ± 1.022 mmol/L. Univariate analysis revealed that, except for diabetes history and alcohol consumption on TC, the impact of dietary Cu level and other covariates on TC was statistically significant.

**Figure 1:**
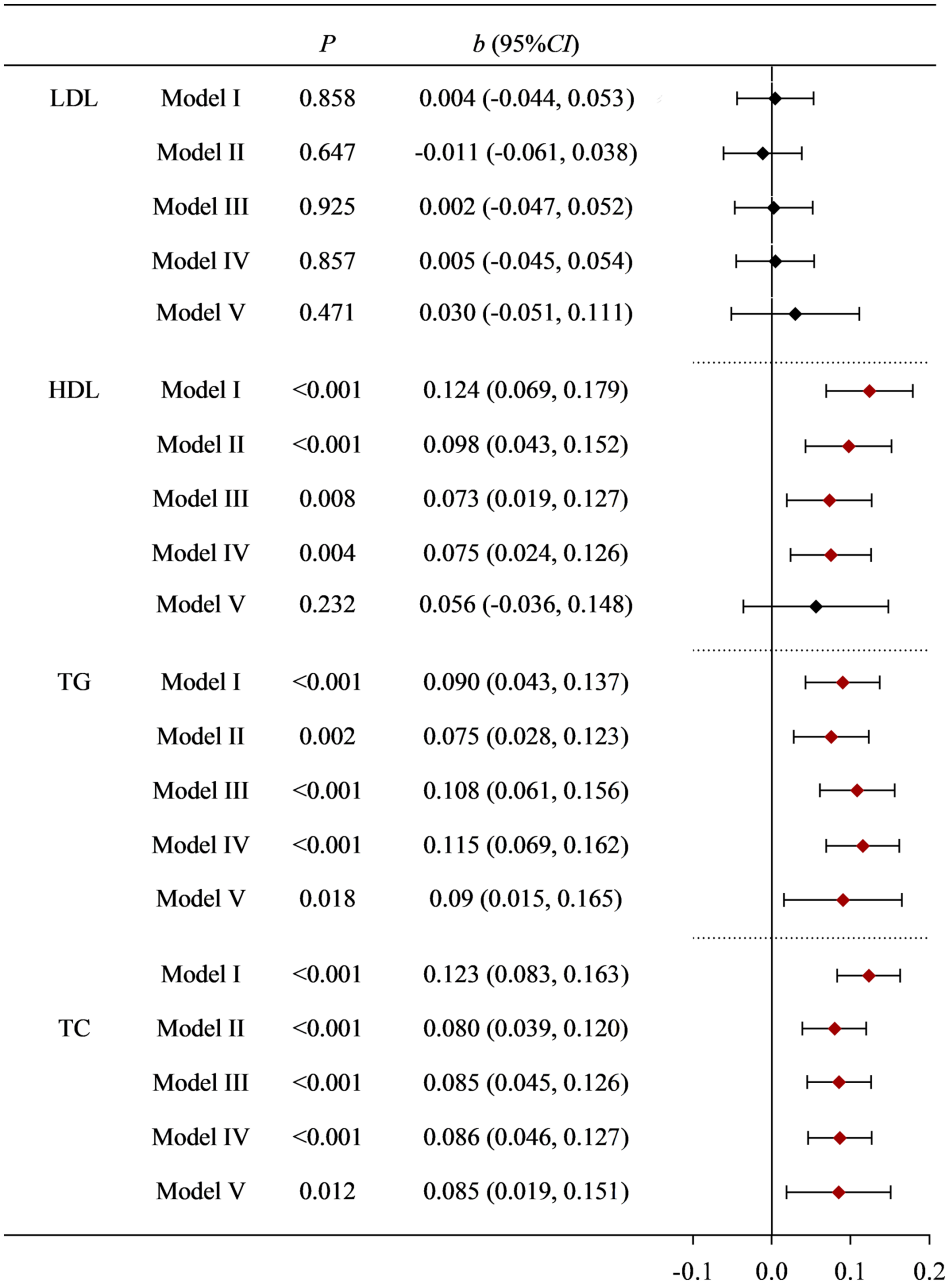
Correlation between dietary Cu intake and blood lipid indicators in NHANES study. Model I: unadjusted. Model II: adjusted for race, education level, country, marital status, and age. Model III: further adjusted for smoking status, drinking status, history of diabetes, history of hypertension, and PA. Model IV: further adjusted for BMI and fast glucose levels. Model V: further adjusted for dietary cholesterol, dietary fiber, and dietary zinc.

Regardless of whether the covariates were adjusted or not, there was no statistical difference between dietary Cu intake levels and LDL. Dietary Cu was not statistically significantly associated with HDL after adjustment with dietary fiber, dietary zinc, and dietary cholesterol, but was associated in the Model I–IV. The positive association between dietary Cu intake and TG, and TC remained stable after multiple adjustments for covariates. See Supplementary Tables S1–S4 and Figure 1 for details.

### 3.2 Correlation between dietary Cu intake and blood lipid indicators in the on-site study

**Figure 2:**
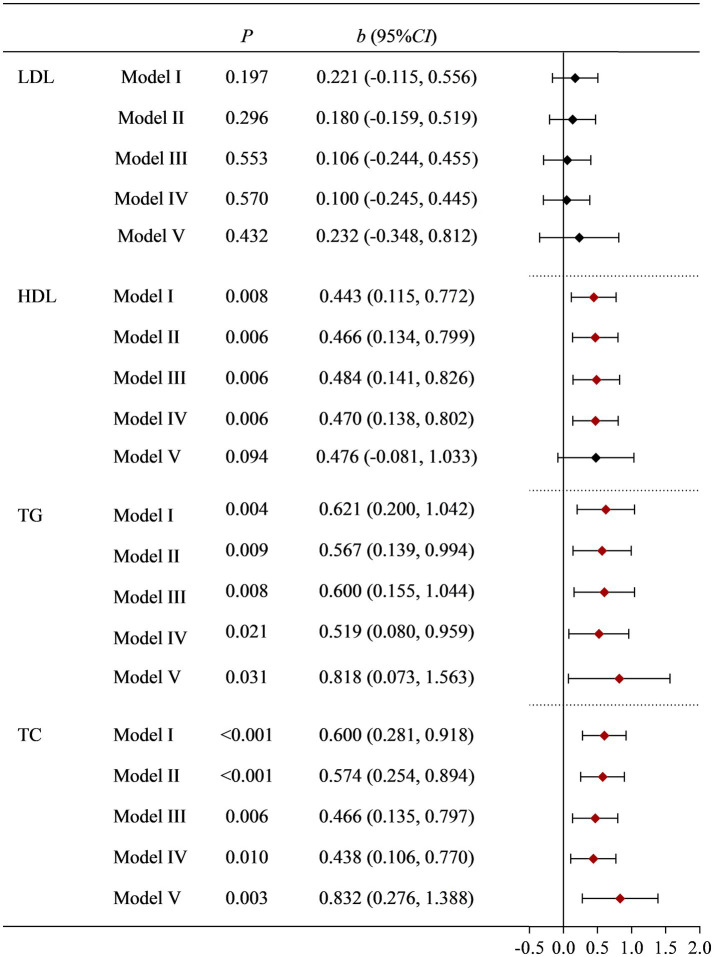
Correlation between dietary Cu intake and blood lipid indicators in the on-site study. Model I: unadjusted. Model II: adjusted for occupation, marital status, and age. Model III: further adjusted for smoking status, drinking status, history of diabetes, history of hypertension, and PA. Model IV: further adjusted for BMI and fast glucose levels. Model V: further adjusted for dietary cholesterol, dietary fiber, and dietary zinc.

To further validate the research results from NHANES, we conducted a survey on women of childbearing age who underwent physical examinations at a tertiary hospital. According to the inclusion and exclusion criteria, a total of 541 eligible research subjects were obtained. The basic information of the research subjects and the effects of various factors on blood lipid indicators (LDL, HDL, TC, and TG) are shown in Supplementary Table S5. Univariate analysis found that age, fast blood glucoses, BMI, occupation, drinking status, and Moderate PA had a statistically significant impact on LDL. The effects of fast blood glucoses, BMI, dietary Cu levels, dietary cholesterol, and history of hypertension on HDL were also statistically significant. Age, BMI, dietary Cu level, dietary cholesterol, dietary zinc, occupation, history of diabetes, and history of hypertension had statistically significant effects on TC. BMI, dietary Cu, and dietary cholesterol levels have statistically significant effects on TG. The positive association between dietary Cu intake and TG, and TC remained stable after multiple adjustments for covariates. See Supplementary Table S5 and Figure 2 for details.

### 3.3 Animal experiments have confirmed that dietary Cu can affect the levels of blood lipid indicators, and the gut microbiota undergo corresponding changes

**Figure 3:**
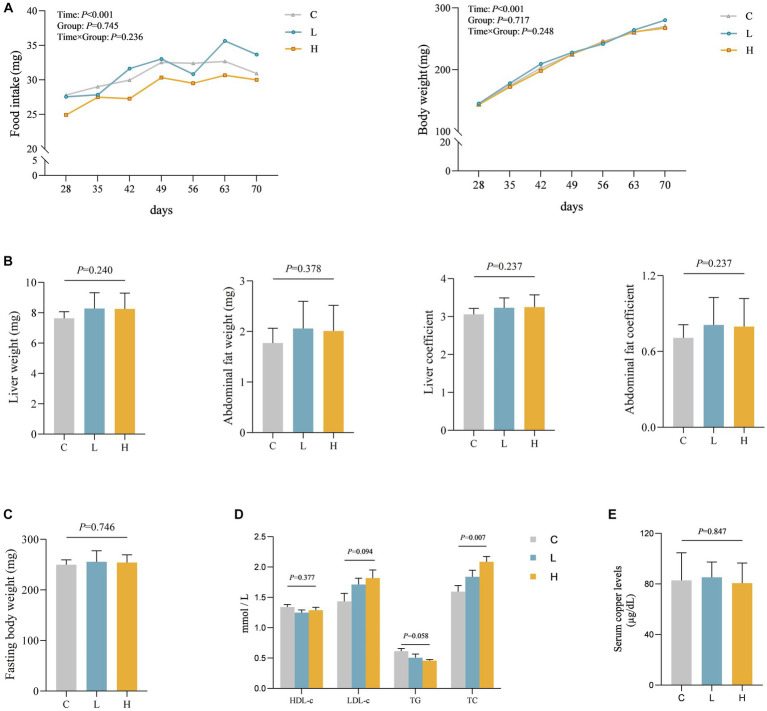
Levels of various indicators during and after intervention in rats (*n* = 9 for each group). **(A)** The food intake and body weight change of rats during the feeding period. **(B–E)** Comparison of organ and organ coefficients **(B)**, fasting body weight **(C)**, blood lipids **(D)**, and blood Cu **(E)** in rats after different doses of Cu intervention. C, Control group; L, Low-dose group; and H, High-dose group.

To verify the relationship between Cu intake and blood lipids, as well as potential influence pathways, we conducted an animal experiment. A total of three groups were made for the animal study, with nine rats in every single group, and no rats died during the study. Aside from the time effect (*p*_time_ < 0.001), there were no statistically significant differences in food intake, weight changes, and interaction between groups and time during the feeding period among the three groups (*p* > 0.05). Moreover, no statistically significant differences were found in liver weight, liver coefficient, abdominal fat weight, abdominal fat coefficient, and fasting weight among the three groups (*p* > 0.05) after intervention. A statistical difference in TC was observed among the three groups, showing a dose–response relationship (*p* = 0.001) where total cholesterol levels increased with increasing dose. However, there was no statistically significant difference in Cu levels in the serum of the three groups of rats (*p* = 0.847). See Figures 3A–E for details.

**Figure 4:**
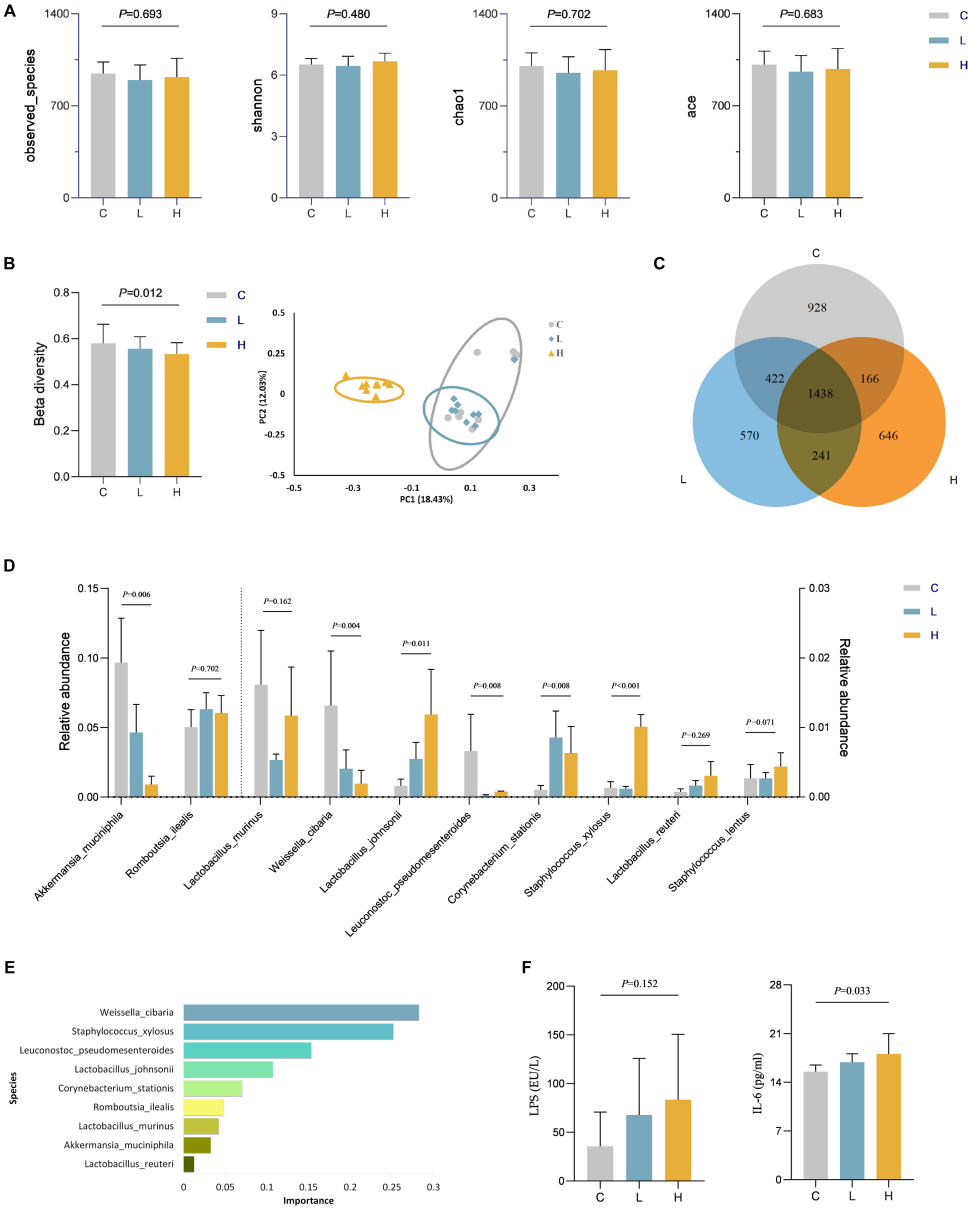
Changes in gut microbiota and levels of LPS and IL-6 in rats after Cu intervention (*n* = 9 for each group). **(A)** Alpha diversity; **(B)** Beta diversity; **(C)** Venn diagram of the OTU distribution; **(D)** Comparison of differences in the top 10 gut microbiota among three groups at the specie level; **(E)** The importance ranking of gut microbiota based on XGBOOST; **(F)** LPS and IL-6 levels in rats after Cu intervention. C, Control group; L, Low-dose group; and H, High-dose group.

We speculate that dietary intake of Cu may play a role through other pathways. Therefore, the level of gut microbiota in rats was assessed. There was no statistically significant difference in alpha diversity among the three groups of rats (Figure 4A). However, ternary phase diagrams analysis (Supplementary Figure S1) of the top 10 gut microbiota at each level revealed that some gut microbiotas were biased toward a certain dose group, indicating that the abundance of some gut microbiota was affected by the intervention. Supplementary Figure S2 showed the relative abundance of gut microbiota at the phylum and genus levels in all animal samples, also indicating alterations in microbial abundance. The results of beta diversity in Figure 4B suggest a statistically significant difference in the composition of gut microbiota among the three groups (*p* = 0.012), and the PcoA plot also indicates that the three groups of microbiotas can be well separated, further confirming the changes in gut microbiota after Cu intervention. Additionally, Figure 4C illustrates a Venn diagram showing unique and shared OTUs among all groups. By combining the importance of XGBOOST microbiota and the Wilcoxon rank sum test analysis, it was found that after intervention, there were statistical differences in the abundance changes of *Weissella cibaria*, *Staphylococcus Xylosus*, *Leuconostoc Pseudosenteroides*, *Lactobacillus Johansonii*, *Corynebacterium Stationis*, and *Akkermansia Muciniphila* in the intestine of rats. The top three most important bacteria were *Weissella cibaria*, *Staphylococcus_Xylosus*, and *Leuconostoc Pseudoteneteroides* (Figures 4D,E). Moreover, the levels of LPS and IL-6 in rats after Cu intervention were increased in the high-dose group, See Figure 4F for details.

## 4 Discussion

This study found that increased dietary Cu intake levels in women of childbearing age can lead to an increase in blood lipid indicators, particularly TC levels. Following the oral intake of Cu, changes were observed in the composition of the intestinal microbiota, notably a significant reduction in the abundance of *Weissella cibaria* and *Leuconostoc pseudoteneteroides*, and a significant increase abundance of *Staphylococcus xylosus*.

In the population surveys, NHANES results were consistent with on-site findings, showing that TG, and TC levels increased with higher dietary Cu intake. Our findings align with the research of Chen et al. and David et al. (10, 19), while inconsistent with the findings of Engle et al. (20). By reviewing the literature, we found that in studies consistent with our results, Cu exposure levels were low: with average Cu intake in our study ranging from 1.18 to 1.8 mg/day, indicating low-dose exposure. Conversely, in studies demonstrating an inverse relationship between Cu intake and blood lipid indicators, Cu intake levels were relatively high. For instance, in Engle’s study, Cu intake for fattening cattle was 10 and 20 mg/kg in the diet (21). Wilson’s disease, a genetic disorder characterized by Cu accumulation and toxicity, exhibits decreased serum triglycerides as one of its typical early pathological changes (22). These results collectively suggest that the lipid metabolism disorder induced by Cu is associated with exposure dose: low-dose Cu exposure leads to increased lipid level, while excessive Cu exposure can result in Cu poisoning, ultimately causing lipid breakdown (manifested as a decrease in total cholesterol and triglyceride levels) (23).

In this study, we found that the participants’ Cu intake levels were at a low dose, which did not exceed the recommended daily intake limit (8 mg/day) for Chinese residents (24). Despite this, we observed a positive relationship between Cu intake and elevated blood lipid levels. To further investigate whether reaching the upper intake limit of Cu (8 mg/day) for Chinese residents will affect blood lipids, we conducted supplementary animal experiments. Although the difference in TG among the three groups was no longer statistically significant, TC still showed a dose–response relationship. We speculate that the lack of statistical significance in TG among the three groups may be attributed to Cu toxicity resulting from the intake of Cu reaching the recommended limit, leading to lipid breakdown. However, the specific mechanism still requires further research.

Through the two population studies, we found that the majority of survey subjects’ Cu intake can meet and exceed the lower limit of the recommended intake, but there is still a certain proportion of study subjects with insufficient Cu intake. This insufficiency may reduce the activity of related enzymes and affect bodily functions. Additionally, based on a large number of population studies and the results of our study, the daily intake of Cu for ordinary people is unlikely to exceed 8 mg/day (25). The recommended maximum tolerance dose for Cu in North America and the European Union is 5 mg/day (26, 27), while the maximum tolerance dose for Cu intake in China is 8 mg/day (24). Our animal experiment revealed that when Cu intake reached 8 mg/day, the study animals tended to experience lipid breakdown related to Cu poisoning. Additionally, the abundance of probiotics related to lipid metabolism decreased, while the abundance of conditional pathogens increased, which is detrimental to health. Therefore, we believe that China’s daily recommended intake limit of 8 mg/d can be appropriately lowered.

Cu absorbed into the bloodstream through the intestines can play an important role in the human body through various pathways (1, 4). Long-term exposure to low-dose Cu can cause lipid aggregation through multiple pathways, such as regulating LXR α, SREBP1, and other lipid synthesis-related genes (28). Additionally, population studies have found that blood Cu levels increase with lower levels of leptin and insulin, and higher BMI (11, 29). While leptin and insulin can regulate blood lipid levels by influencing fatty acid metabolism, energy metabolism, and other pathways (30, 31), the association between Cu and leptin, insulin, BMI, as well as the correlation between leptin, insulin, and blood lipid indicators, suggests a possible pathway for Cu to affect blood lipids. Moreover, recent studies have shown that excessive Cu can influence the body through the targeted tricarboxylic acid cycle pathway (also known as “cuproptosis”) (32, 33), which may be another pathway for Cu to affect blood lipids. In these studies, the levels of internally exposed Cu varied among each group. However, in our study, we found no statistically significant difference in serum Cu levels after different low doses of Cu intake (Figure 3E). Thus, we speculated that Cu affects blood lipid metabolism through other pathways.

Literature evidence has indicated that metal ingestion can affect the gut microbiota (14, 15, 34). Therefore, we speculate that oral intake of Cu interacts with the gut microbiota, which in turn influences the host health. Based on this, the study further examined the level of gut microbiota after Cu exposure. We screened the top 10 bacterial genera at various levels for analysis and found that Cu intervention altered the abundance of gut microbiota in animals. XGBOOST combined with Wilcoxon rank-sum test analysis were used to screen the gut microbiota with significant impacts on the body. And results found that *Weissella cibaria* had the greatest impact on health, followed by *Staphylococcus xylosus* and *Leuconostoc pseudoteneteroides*. *Staphylococcus xylosus* and *Leuconostoc pseudomonas* are opportunistic pathogens that can cause infection only in certain situations such as trauma, surgery, or immunosuppression (35, 36). *Weissella cibaria* is a newly discovered Gram-positive bacterium belonging to the Lactobacillus family, widely present in various naturally fermented foods (37). Current studies have evaluated the safety of *Weissella cibaria* and reported its potential as a probiotic, such as antioxidant, immune regulation, and production of extracellular polysaccharides (38–40). Additionally, research has also found that *Weissella cibaria* can inhibit the production of LPS by harmful bacteria, alleviate the body’s inflammatory response by reducing the levels of inflammatory factors such as IL-6, and improve glucose and lipid metabolism (41). Given the biological functions of the aforementioned bacteria, we speculate that Cu intake alters the homeostasis of the gut microbiota, particularly by reducing the abundance of *Weissella cibaria*, leading to an increase in LPS levels, resulting in elevated IL-6 inflammation levels and ultimately affecting blood lipid levels. Therefore, we further analyzed the levels of LPS and IL-6 in rats after Cu intervention, and the increase in LPS and IL-6 levels in the high-dose Cu intake group confirmed our hypothesis. The study of gut microbiota suggests that regulating gut microbiota, especially supplementing probiotics, may be another direction for regulating lipid metabolism.

Our study integrates NHANES population data, on-site research, and animal experiments to comprehensively examine the link between dietary Cu intake and blood lipids. The alignment of NHANES data with on-site study outcomes strengthens this association, and animal experiments further substantiate the influence of Cu on lipid levels. Furthermore, our exploration of intestinal flora, including the impact of Cu intake on specific microbes such as *Weissella cibaria*, provides valuable insights for potential interventions in lipid metabolism. However, our study still has the following limitations. First, our study identified correlations between Cu intake and blood lipids through population research. Although we adopted multiple statistical models to control for potential factors, we must acknowledge that we cannot eliminate all possible confounding factors. While animal experiments have provided further evidence of Cu intake affecting blood lipid levels, it is important to note that animal models cannot fully replicate human physiology. Second, our study mainly focused on women of childbearing age and validated the findings through two distinct data sources: the NHANES database and on-site study. However, dietary habits and cultural factors in different regions may introduce certain influences on the results. Therefore, the extrapolation of the findings of our study might be limited. Third, our study only focused on the differences in gut microbiota after Cu intervention to reflect the changes in gut microbiota following prolonged Cu intake. By focusing solely on post-intervention changes, we did not capture the long-term impacts or dynamic alterations in gut microbiota. Future research could consider delving into the dynamic changes in gut microbiota following Cu exposure from both human and animal perspectives, thereby providing a more in-depth scientific basis for the association between Cu and blood lipids. Finally, the quality and accuracy of NHANES data and on-site survey data have a significant impact on result reliability, particularly influenced by recall bias. Despite our efforts during on-site investigations to minimize these biases and ensure data accuracy through multiple questionnaire optimizations, as well as the engagement of experienced surveyors, factors like recall bias may persist. Therefore, further validation through a multi-center, population-based experimental study is essential for future research.

## 5 Conclusion

The results of this study suggest that for women of childbearing age, as daily Cu intake increases, blood lipid levels also increase. From the perspective of Cu intake levels among women of childbearing age, nutrition-related departments of China government can consider appropriately reducing the upper limit of recommended daily Cu intake for women of childbearing age. In addition, regulating the gut microbiota, especially supplementing *Weissella cibaria*, may be another effective way to intervene in blood lipids.

## Data availability statement

The data presented in the study are deposited in the European Nucleotide Archive (ENA) at EMBL-EBI repository, accession number PRJEB72719.

## Ethics statement

The studies involving humans were approved by the Ethics Committee of Gannan Medical University. The studies were conducted in accordance with the local legislation and institutional requirements. The participants provided their written informed consent to participate in this study. The animal study was approved by the Ethics Committee of Gannan Medical University. The study was conducted in accordance with the local legislation and institutional requirements.

## Author contributions

ML: Project administration, Writing – original draft, Writing – review & editing, Formal Analysis, Methodology. LG: Methodology, Writing – review & editing, Data curation, Investigation, Validation. CW: Writing – review & editing, Conceptualization, Methodology, Supervision. MH: Supervision, Validation, Writing – review & editing. JG: Validation, Methodology, Writing – review & editing. XL: Data curation, Investigation, Writing – review & editing. QW: Conceptualization, Funding acquisition, Methodology, Project administration, Supervision, Writing – review & editing.

## References

[1] Kim B Nevitt T Thiele D. Mechanisms for copper acquisition, distribution and regulation. Nat Chem Biol. (2008) 4:176–85. doi: 10.1038/nchembio.72, PMID: 18277979

[2] Li J Yin J Li W Wang H Ni B. Molecular subtypes based on cuproptosis-related genes and tumor microenvironment infiltration characteristics in pancreatic adenocarcinoma. Cancer Cell Int. (2023) 23:7. doi: 10.1186/s12935-022-02836-z, PMID: 36647100 PMC9844034

[3] Ranjan P Ghosh D Yarramala D Das S Maji S Kumar A. Differential copper binding to alpha-synuclein and its disease-associated mutants affect the aggregation and amyloid formation. Biochim Biophys Acta, Gen Subj. (2017) 1861:365–74. doi: 10.1016/j.bbagen.2016.11.043, PMID: 27916677

[4] Turski M Thiele D. New roles for copper metabolism in cell proliferation, signaling, and disease. J Biol Chem. (2009) 284:717–21. doi: 10.1074/jbc.R800055200, PMID: 18757361 PMC2613604

[5] Wooton-Kee CR Robertson M Zhou Y Dong B Sun Z Kim KH. Metabolic dysregulation in the Atp7b (−/−) Wilson's disease mouse model. Proc Natl Acad Sci. (2020) 117:2076–83. doi: 10.1073/pnas.1914267117, PMID: 31924743 PMC6994990

[6] Zhang B He M. Identification of potential biomarkers for coronary artery disease based on Cuproptosis. Cardiovasc Ther. (2023) 2023:5996144–13. doi: 10.1155/2023/5996144, PMID: 36743388 PMC9891837

[7] Kunutsor S Dey R Laukkanen J. Circulating serum copper is associated with atherosclerotic cardiovascular disease, but not venous thromboembolism: a prospective cohort study. Pulse. (2021) 9:109–15. doi: 10.1159/000519906, PMID: 35083177 PMC8739646

[8] Yang H Ralle M Wolfgang M Dhawan N Burkhead J Rodriguez S. Copper-dependent amino oxidase 3 governs selection of metabolic fuels in adipocytes. PLoS Biol. (2018) 16:e2006519. doi: 10.1371/journal.pbio.2006519, PMID: 30199530 PMC6130853

[9] Lei L Xiaoyi S Fuchang L. Effect of dietary copper addition on lipid metabolism in rabbits. Food Nutr Res. (2017) 61:1348866. doi: 10.1080/16546628.2017.1348866, PMID: 28747869 PMC5510220

[10] Kennedy D Lyn R Pezacki J. Cellular lipid metabolism is influenced by the coordination environment of copper. J Am Chem Soc. (2009) 131:2444–5. doi: 10.1021/ja809451w, PMID: 19187018

[11] Yang H Liu C Wolf R Ralle M Dev S Pierson H. Obesity is associated with copper elevation in serum and tissues. Metallomics. (2019) 11:1363–71. doi: 10.1039/c9mt00148d, PMID: 31249997 PMC7753954

[12] Scheidl T Brightwell A Easson S Thompson J. Maternal obesity and programming of metabolic syndrome in the offspring: searching for mechanisms in the adipocyte progenitor pool. BMC Med. (2023) 21:50. doi: 10.1186/s12916-023-02730-z, PMID: 36782211 PMC9924890

[13] Whitman W Coleman D Wiebe W. Prokaryotes: the unseen majority. Proc Natl Acad Sci. (1998) 95:6578–83. doi: 10.1073/pnas.95.12.6578, PMID: 9618454 PMC33863

[14] Richardson J Dancy B Horton C Lee Y Madejczyk M Xu Z. Exposure to toxic metals triggers unique responses from the rat gut microbiota. Sci Rep. (2018) 8:6578. doi: 10.1038/s41598-018-24931-w, PMID: 29700420 PMC5919903

[15] Li A Ding J Shen T Han Z Zhang J Abadeen Z. Environmental hexavalent chromium exposure induces gut microbial dysbiosis in chickens. Ecotoxicol Environ Saf. (2021) 227:112871. doi: 10.1016/j.ecoenv.2021.112871, PMID: 34649138

[16] Guan X Sun Z. The role of intestinal Flora and its metabolites in heart failure. Infect Drug Resis. (2023) 16:51–64. doi: 10.2147/idr.S390582, PMID: 36636378 PMC9830706

[17] National Institute for Nutrition and Health, Chinese Center for Disease Control and Prevention. China Food Composition Tables. 6th ed Beijing, China: Peking University Medical Press (2019).

[18] Shi X. Modern Medical Experimental Zoology. Beijing: People's Military Doctor Publishing House (2000).

[19] Chen F Luo Z Chen G Shi X Liu X Song Y. Effects of waterborne cu exposure on intestinal copper transport and lipid metabolism of *Synechogobius hasta*. Aquat Toxicol. (2016) 178:171–81. doi: 10.1016/j.aquatox.2016.08.001, PMID: 27509383

[20] Engle T Spears J. Dietary copper effects on lipid metabolism, performance, and ruminal fermentation in finishing steers. J Anim Sci. (2000) 78:2452–8. doi: 10.2527/2000.7892452x, PMID: 10985421

[21] Engle T. Copper and lipid metabolism in beef cattle: a review. J Anim Sci. (2011) 89:591–6. doi: 10.2527/jas.2010-3395, PMID: 20935142

[22] Zhi Y Sun Y Jiao Y Pan C Wu Z Liu C. HR-MS based untargeted Lipidomics reveals characteristic lipid signatures of Wilson's disease. Front Pharmacol. (2021) 12:754185. doi: 10.3389/fphar.2021.754185, PMID: 34880754 PMC8645799

[23] Huster D Purnat T Burkhead J Ralle M Fiehn O Stuckert F. High copper selectively alters lipid metabolism and cell cycle machinery in the mouse model of Wilson disease. J Biol Chem. (2007) 282:8343–55. doi: 10.1074/jbc.M607496200, PMID: 17205981

[24] National Health Commission of People's Republic of China (2017). Chinese dietary reference intakes-Part 3: Trace element.

[25] Klevay L. Is the Western diet adequate in copper? J Trace Element Med Biol. (2011) 25:204–12. doi: 10.1016/j.jtemb.2011.08.146, PMID: 21982501

[26] Nordic Council of Ministers (2014). Nordic Nutrition Recommendations 2012. Edited by Nordic Council of Ministers and Copenhagen D.

[27] European Commission (2003). Opinion of the scientific committee on food on the tolerable upper intake level of copper. Brussels BEc.

[28] Pan Y Zhuo M Li D Xu Y Wu K Luo Z. SREBP-1 and LXRα pathways mediated cu-induced hepatic lipid metabolism in zebrafish *Danio rerio*. Chemosphere. (2019) 215:370–9. doi: 10.1016/j.chemosphere.2018.10.058, PMID: 30336314

[29] Olusi S Al-Awadhi A Abiaka C Abraham M George S. Serum copper levels and not zinc are positively associated with serum leptin concentrations in the healthy adult population. Biol Trace Elem Res. (2003) 91:137–44. doi: 10.1385/bter:91:2:137, PMID: 12719608

[30] Bahrami E Mirmoghtadaee P Ardalan G Zarkesh-Esfahani H Tajaddini M Haghjooy-Javanmard S. Insulin and leptin levels in overweight and normal-weight Iranian adolescents: the CASPIAN-III study. J Res Med Sci. (2014) 19:387–90. PMID: 25097618 PMC4116567

[31] Baskin D Figlewicz Lattemann D Seeley R Woods S Porte D Schwartz M. Insulin and leptin: dual adiposity signals to the brain for the regulation of food intake and body weight. Brain Res. (1999) 848:114–23. doi: 10.1016/s0006-8993(99)01974-5, PMID: 10612703

[32] Tsvetkov P Coy S Petrova B Dreishpoon M Verma A Abdusamad M. Copper induces cell death by targeting lipoylated TCA cycle proteins. Science (New York, NY). (2022) 375:1254–61. doi: 10.1126/science.abf0529 PMC9273333 35298263

[33] Li S Bu L Cai L. Cuproptosis: lipoylated TCA cycle proteins-mediated novel cell death pathway. Signal Transduct Target Therap. (2022) 7:158. doi: 10.1038/s41392-022-01014-x, PMID: 35562341 PMC9106713

[34] Kaur R Rawal R. Influence of heavy metal exposure on gut microbiota: recent advances. J Biochem Mol Toxicol. (2023) 37:e23485. doi: 10.1002/jbt.23485, PMID: 37593904 PMC13546941

[35] Battaglia M Garrett-Sinha L. Staphylococcus xylosus and *Staphylococcus aureus* as commensals and pathogens on murine skin. Lab Anim Res. (2023) 39:18. doi: 10.1186/s42826-023-00169-0, PMID: 37533118 PMC10394794

[36] Cappelli E Barros R Camello T Teixeira L Merquior V. *Leuconostoc pseudomesenteroides* as a cause of nosocomial urinary tract infections. J Clin Microbiol. (1999) 37:4124–6. doi: 10.1128/jcm.37.12.4124-4126.1999, PMID: 10565942 PMC85896

[37] Björkroth K Schillinger U Geisen R Weiss N Hoste B Holzapfel W. Taxonomic study of Weissella confusa and description of *Weissella cibaria* sp. nov., detected in food and clinical samples. Int J Syst Evol Microbiol. (2002) 52:141–8. doi: 10.1099/00207713-52-1-141, PMID: 11837296

[38] Yu H Lee N Choi A Choe J Bae C Paik H. *Weissella cibaria* antagonistic and antioxidant effect of probiotic JW15. Food Sci Biotechnol. (2019) 28:851–5. doi: 10.1007/s10068-018-0519-6, PMID: 31093443 PMC6484072

[39] Park H Kang K Kim B Lee S Lee W. Immunomodulatory potential of *Weissella cibaria* in aged C57BL/6J mice. J Microbiol Biotechnol. (2017) 27:2094–103. doi: 10.4014/jmb.1708.08016, PMID: 29032650

[40] Park S Saravanakumar K Sathiyaseelan A Han K Lee J Wang M. Polysaccharides of *Weissella cibaria* act as a prebiotic to enhance the probiotic potential of *Lactobacillus rhamnosus*. Appl Biochem Biotechnol. (2023) 195:3928–40. doi: 10.1007/s12010-022-04104-2, PMID: 35947292

[41] Xie Y Pei F Liu Y Liu Z Chen X Xue D. Fecal fermentation and high-fat diet-induced obesity mouse model confirmed exopolysaccharide from *Weissella cibaria* PFY06 can ameliorate obesity by regulating the gut microbiota. Carbohydr Polym. (2023) 318:121122. doi: 10.1016/j.carbpol.2023.121122, PMID: 37479437

