# Supplementary material for: Effects of dietary copper intake on blood lipids in women of childbearing age and the potential role of gut microbiota
Source: Front Nutr. 2024 Mar 5;11:1368730. doi: 10.3389/fnut.2024.1368730 (PMC10948407; doi:10.3389/fnut.2024.1368730)
Supplement: Supplementary file 1 [file Data_Sheet_1.docx]

**Table S1** Correlation analysis between LDL level and other indexes (*n*=5326)

|  |  | $\bar{x}\pm S$ / *n*(%) | *P* | *b* (95%*CI*) |
| --- | --- | --- | --- | --- |
| LDL (mmol/L) | | 2.789 ± 0.848 | - | - |
| Age (years) | | 34.182 ± 8.565 | <0.001 | 0.018 (0.016, 0.021) |
| Glucoses (mmol/l) | | 5.133 ± 1.338 | <0.001 | 0.052 (0.035, 0.069) |
| BMI (kg/m^2^) | | 29.453 ± 8.074 | <0.001 | 0.013 (0.010, 0.016) |
| Dietary Cu (mg) | | 1.122 ± 0.659 | 0.858 | 0.004 (-0.044, 0.053) |
| Dietary cholesterol (mg) | | 266.119 ± 211.034 | 0.121 | 0.018 (-0.005, 0.041) |
| Dietary fiber (gm) | | 14.930 ± 9.214 | 0.875 | 0.003 (-0.031, 0.036) |
| Dietary zinc (mg) | | 9.842 ± 5.661 | 0.322 | 0.020 (-0.020, 0.060) |
| Education | high school graduate/ged or equivalent | 1027 (19.28) | ref | ref |
|  | 9-11th grade (includes 12th grade with no diploma) | 682 (12.81) | 0.083 | 0.073 (-0.01, 0.155) |
|  | college graduate or above | 1409 (26.45) | 0.436 | -0.027 (-0.095, 0.041) |
|  | less than 9th grade | 350 (6.57) | 0.009 | 0.137 (0.034, 0.240) |
|  | some college or aa degree | 1858 (34.89) | 0.854 | 0.006 (-0.059, 0.071) |
| Race | Mexican American | 976 (18.33) | ref | ref |
|  | non-Hispanic black | 1207 (22.66) | 0.014 | -0.089 (-0.161, -0.018) |
|  | non-Hispanic white | 1975 (37.08) | 0.542 | 0.020 (-0.045, 0.085) |
|  | other Hispanic | 481 (9.03) | 0.758 | -0.015 (-0.107, 0.078) |
|  | other race - including multi-racial | 687 (12.9) | 0.041 | -0.086 (-0.169, -0.003) |
| Country | born elsewhere | 102 (1.92) | ref | ref |
|  | born in 50 U.S. states or Washington, dc | 3812 (71.57) | 0.997 | <0.001 (-0.166, 0.167) |
|  | born in Mexico | 335 (6.29) | 0.085 | 0.165 (-0.023, 0.353) |
|  | born in other non-Spanish speaking country | 85 (1.60) | 0.620 | -0.062 (-0.306, 0.182) |
|  | born in other Spanish speaking country | 103 (1.93) | 0.024 | 0.266 (0.034, 0.498) |
|  | others | 889 (16.69) | 0.623 | 0.043 (-0.130, 0.217) |
| History of diabetes | borderline | 67 (1.26) | ref | ref |
|  | no | 5030 (94.44) | 0.473 | -0.075 (-0.279, 0.130) |
|  | yes | 229 (4.3) | 0.562 | 0.068 (-0.163, 0.299) |
| History of hypertension | no | 4461 (83.76) | ref | ref |
|  | yes | 865 (16.24) | 0.009 | 0.083 (0.021, 0.144) |
| Smoke status | every day | 2312 (43.41) | ref | ref |
|  | not at all | 2254 (42.32) | 0.629 | -0.012 (-0.061, 0.037) |
|  | some days | 760 (14.27) | 0.002 | -0.112 (-0.181, -0.042) |
| Vigorous PA | no | 3850 (72.29) | ref | ref |
|  | unable to do activity | 19 (0.36) | 0.700 | 0.075 (-0.306, 0.456) |
|  | yes | 1457 (27.35) | <0.001 | -0.153 (-0.204, -0.102) |
| Moderate PA | no | 2831 (53.15) | ref | ref |
|  | unable to do activity | 12 (0.23) | 0.728 | 0.085 (-0.396, 0.566) |
|  | yes | 2483 (46.62) | 0.308 | -0.024 (-0.07, 0.022) |
| Marital status | married/living with partner | 3170 (59.52) | ref | ref |
|  | widowed/divorced/separated | 690 (12.95) | 0.236 | 0.042 (-0.027, 0.111) |
|  | never married | 1466 (27.53) | <0.001 | -0.203 (-0.256, -0.151) |
| Alcohol status | not drinking | 4299 (80.72) | ref | ref |
|  | drinking | 1027 (19.28) | <0.001 | -0.107 (-0.164, -0.049) |

**Table S2** Correlation analysis between HDL level and other indexes (*n*=1072)

|  |  | $\bar{x}\pm S$ / *n*(%) | *P* | *b* (95%*CI*) |
| --- | --- | --- | --- | --- |
| HDL (mmol/L) | | 1.538 ± 0.430 | - | - |
| Age (years) | | 33.55 ± 8.594 | 0.006 | -0.004 (-0.007, -0.001) |
| Glucoses (mmol/l) | | 4.838 ± 0.918 | <0.001 | -0.112 (-0.139, -0.085) |
| BMI (kg/m^2^) | | 28.936 ± 7.447 | <0.001 | -0.017 (-0.020, -0.013) |
| Dietary Cu (mg) | | 1.149 ± 0.641 | <0.001 | 0.124 (0.069, 0.179) |
| Dietary cholesterol (mg) | | 271.743 ± 216.338 | 0.052 | 0.030 (0.000, 0.060) |
| Dietary fiber (gm) | | 14.238 ± 8.329 | 0.004 | 0.060 (0.019, 0.101) |
| Dietary zinc (mg) | | 11.043 ± 7.526 | 0.004 | 0.065 (0.020, 0.109) |
| Education | high school graduate/ged or equivalent | 247 (23.04) | ref | ref |
|  | 9-11th grade (includes 12th grade with no diploma) | 169 (15.77) | 0.601 | 0.022 (-0.060, 0.104) |
|  | college graduate or above | 241 (22.48) | <0.001 | 0.272 (0.198, 0.346) |
|  | less than 9th grade | 68 (6.34) | 0.758 | 0.018 (-0.095, 0.130) |
|  | some college or aa degree | 347 (32.37) | 0.001 | 0.117 (0.049, 0.185) |
| Race | Mexican American | 236 (22.01) | ref | ref |
|  | non-Hispanic black | 236 (22.01) | 0.146 | 0.057 (-0.020, 0.135) |
|  | non-Hispanic white | 517 (48.23) | 0.001 | 0.108 (0.042, 0.174) |
|  | other Hispanic | 40 (3.73) | 0.451 | -0.055 (-0.199, 0.088) |
|  | other race - including multi-racial | 43 (4.01) | 0.012 | 0.179 (0.040, 0.318) |
| Country | born elsewhere | 93 (8.67) | ref | ref |
|  | born in 50 U.S. states or Washington, dc | 834 (77.8) | 0.285 | -0.050 (-0.142, 0.042) |
|  | born in Mexico | 145 (13.53) | 0.005 | -0.159 (-0.270, -0.047) |
| History of  diabetes | borderline | 6 (0.56) | ref | ref |
|  | no | 1032 (96.27) | 0.702 | 0.067 (-0.277, 0.412) |
|  | yes | 34 (3.17) | 0.448 | -0.144 (-0.517, 0.228) |
| History of  hypertension | no | 905 (84.42) | ref | ref |
|  | yes | 167 (15.58) | <0.001 | -0.134 (-0.205, -0.064) |
| Smoke status | every day | 469 (43.75) | ref | ref |
|  | not at all | 465 (43.38) | <0.001 | 0.181 (0.127, 0.235) |
|  | some days | 138 (12.87) | <0.001 | 0.151 (0.071, 0.231) |
| Vigorous PA | no | 728 (67.91) | ref | ref |
|  | unable to do activity | 20 (1.87) | 0.693 | -0.039 (-0.230, 0.153) |
|  | yes | 324 (30.22) | 0.082 | 0.050 (-0.006, 0.106) |
| Moderate PA | no | 452 (42.16) | ref | ref |
|  | unable to do activity | 11 (1.03) | 0.919 | -0.013 (-0.270, 0.243) |
|  | yes | 609 (56.81) | 0.002 | 0.081 (0.029, 0.133) |
| Marital status | married/living with partner | 654 (61.01) | ref | ref |
|  | widowed/divorced/separated | 162 (15.11) | 0.006 | -0.104 (-0.177, -0.030) |
|  | never married | 256 (23.88) | 0.074 | -0.056 (-0.118, 0.006) |
| Alcohol status | not drinking | 856 (79.85) | ref | ref |
|  | drinking | 216 (20.15) | <0.001 | 0.136 (0.072, 0.200) |

**Table S3** Correlation analysis between TG level and other indexes (*n*=11271)

|  |  | $\bar{x}\pm S$ / *n*(%) | *P* | *b* (95%*CI*) |
| --- | --- | --- | --- | --- |
| TG (mmol/L) | | 1.385 ± 1.196 |  |  |
| Age (years) | | 34.249 ± 8.616 | <0.001 | 0.015 (0.013, 0.018) |
| Glucoses (mmol/l) | | 5.128 ± 1.543 | <0.001 | 0.162 (0.148, 0.176) |
| BMI (kg/m^2^) | | 29.462 ± 8.026 | <0.001 | 0.026 (0.023, 0.029) |
| Dietary Cu (mg) | | 1.134 ± 0.696 | <0.001 | 0.090 (0.043, 0.137) |
| Dietary cholesterol (mg) | | 265.630 ± 214.499 | 0.281 | 0.012 (-0.01, 0.035) |
| Dietary fiber (gm) | | 15.044 ± 9.379 | <0.001 | 0.077 (0.044, 0.109) |
| Dietary zinc (mg) | | 9.906 ±6.052 | <0.001 | 0.079 (0.040, 0.118) |
| Education | high school graduate/ged or equivalent | 2251 (19.97) | ref | ref |
|  | 9-11th grade (includes 12th grade with no diploma) | 1468 (13.02) | <0.001 | 0.048 (-0.031, 0.126) |
|  | college graduate or above | 2891 (25.65) | <0.001 | -0.244 (-0.309, -0.178) |
|  | less than 9th grade | 711 (6.31) | <0.001 | 0.289 (0.189, 0.389) |
|  | some college or aa degree | 3950 (35.05) | <0.001 | -0.124 (-0.185, -0.062) |
| Race | Mexican American | 2095 (18.59) | ref | ref |
|  | non-Hispanic black | 2528 (22.43) | <0.001 | -0.593 (-0.662, -0.525) |
|  | non-Hispanic white | 4213 (37.38) | <0.001 | -0.185 (-0.247, -0.123) |
|  | other Hispanic | 1054 (9.35) | <0.001 | -0.162 (-0.250, -0.075) |
|  | other race - including multi-racial | 1381 (12.25) | <0.001 | -0.231 (-0.312, -0.151) |
| Country | born elsewhere | 216 (1.92) | ref | ref |
|  | born in 50 U.S. states or Washington, dc | 8070 (71.6) | 0.529 | 0.052 (-0.109, 0.213) |
|  | born in Mexico | 697 (6.18) | <0.001 | 0.430 (0.249, 0.612) |
|  | born in other non-Spanish speaking country | 176 (1.56) | 0.586 | 0.066 (-0.171, 0.303) |
|  | born in other Spanish speaking country | 213 (1.89) | 0.525 | 0.073 (-0.152, 0.299) |
|  | others | 1899 (16.85) | 0.004 | 0.247 (0.079, 0.415) |
| History of  diabetes | borderline | 136 (1.21) | ref | ref |
|  | no | 10642 (94.42) | 0.001 | -0.342 (-0.542, -0.142) |
|  | yes | 493 (4.37) | <0.001 | 0.499 (0.274, 0.723) |
| History of  hypertension | no | 9501 (84.3) | ref | ref |
|  | yes | 1770 (15.7) | <0.001 | 0.322 (0.262, 0.383) |
| Smoke status | every day | 4816 (42.73) | ref | ref |
|  | not at all | 4740 (42.05) | 0.927 | 0.002 (-0.046, 0.050) |
|  | some days | 1715 (15.22) | 0.153 | -0.048 (-0.114, 0.018) |
| Vigorous PA | no | 8108 (71.94) | ref | ref |
|  | unable to do activity | 42 (0.37) | 0.394 | 0.157 (-0.204, 0.518) |
|  | yes | 3121 (27.69) | <0.001 | -0.282 (-0.331, -0.233) |
| Moderate PA | no | 5978 (53.04) | ref | ref |
|  | unable to do activity | 24 (0.21) | 0.575 | 0.137 (-0.342, 0.616) |
|  | yes | 5269 (46.75) | <0.001 | -0.116 (-0.161, -0.072) |
| Marital status | married/living with partner | 6714 (59.57) | ref | ref |
|  | widowed/divorced/separated | 1464 (12.99) | 0.161 | 0.048 (-0.019, 0.115) |
|  | never married | 3093 (27.44) | <0.001 | -0.282 (-0.333, -0.231) |
| Alcohol status | not drinking | 9064 (80.42) | ref | ref |
|  | drinking | 2207 (19.58) | <0.001 | -0.153 (-0.209, -0.098) |

**Table S4** Correlation analysis between TC level and other indexes (*n*=11279)

|  |  | $\bar{x}\pm S$ / *n*(%) | *P* | *b* (95%*CI*) |
| --- | --- | --- | --- | --- |
| TC (mmol/L) | | 4.89 ± 1.022 |  |  |
| Age (years) | | 34.245 ± 8.617 | <0.001 | 0.022 (0.02, 0.024) |
| Glucoses (mmol/l) | | 5.128 ± 1.542 | <0.001 | 0.049 (0.037, 0.061) |
| BMI (kg/m^2^) | | 29.46 ± 8.028 | <0.001 | 0.005 (0.003, 0.008) |
| Dietary Cu (mg) | | 1.134 ± 0.696 | <0.001 | 0.123 (0.083, 0.163) |
| Dietary cholesterol (mg) | | 265.640 ± 214.481 | 0.008 | 0.026 (0.007, 0.046) |
| Dietary fiber (gm) | | 15.045 ± 9.377 | <0.001 | 0.052 (0.024, 0.080) |
| Dietary zinc (mg) | | 9.907 ±6.050 | <0.001 | 0.080 (0.047, 0.113) |
| Education | high school graduate/ged or equivalent | 2255 (19.99) | ref | ref |
|  | 9-11th grade (includes 12th grade with no diploma) | 1469 (13.03) | 0.432 | 0.027 (-0.04, 0.094) |
|  | college graduate or above | 2893 (25.65) | 0.084 | 0.05 (-0.007, 0.106) |
|  | less than 9th grade | 712 (6.31) | <0.001 | 0.156 (0.07, 0.243) |
|  | some college or aa degree | 3950 (35.02) | 0.834 | -0.006 (-0.058, 0.047) |
| Race | Mexican American | 2097 (18.59) | ref | ref |
|  | non-Hispanic black | 2529 (22.42) | <0.001 | -0.263 (-0.322, -0.205) |
|  | non-Hispanic white | 4216 (37.38) | 0.008 | 0.072 (0.019, 0.125) |
|  | other Hispanic | 1055 (9.36) | 0.081 | -0.067 (-0.142, 0.008) |
|  | other race - including multi-racial | 1382 (12.25) | 0.002 | -0.107 (-0.176, -0.038) |
| Country | born elsewhere | 216 (1.92) | ref | ref |
|  | born in 50 U.S. states or Washington, dc | 8075 (71.59) | 0.004 | -0.2 (-0.338, -0.062) |
|  | born in Mexico | 697 (6.18) | 0.345 | 0.075 (-0.081, 0.231) |
|  | born in other non-Spanish speaking country | 178 (1.58) | 0.056 | -0.197 (-0.399, 0.005) |
|  | born in other Spanish speaking country | 212 (1.88) | 0.409 | -0.081 (-0.275, 0.112) |
|  | others | 1901 (16.85) | 0.002 | -0.23 (-0.373, -0.086) |
| History of  diabetes | borderline | 136 (1.21) | ref | ref |
|  | no | 10649 (94.41) | 0.368 | -0.079 (-0.252, 0.093) |
|  | yes | 494 (4.38) | 0.164 | 0.137 (-0.056, 0.331) |
| History of  hypertension | no | 9502 (84.25) | ref | ref |
|  | yes | 1777 (15.75) | <0.001 | 0.116 (0.064, 0.167) |
| Smoke status | every day | 4801 (42.57) | ref | ref |
|  | not at all | 4768 (42.27) | <0.001 | 0.111 (0.071, 0.152) |
|  | some days | 1710 (15.16) | 0.304 | -0.03 (-0.086, 0.027) |
| Vigorous PA | no | 8116 (71.96) | ref | ref |
|  | unable to do activity | 42 (0.37) | 0.001 | 0.514 (0.205, 0.823) |
|  | yes | 3121 (27.67) | <0.001 | -0.133 (-0.176, -0.091) |
| Moderate PA | no | 5982 (53.04) | ref | ref |
|  | unable to do activity | 24 (0.21) | 0.047 | 0.416 (0.006, 0.826) |
|  | yes | 5273 (46.75) | 0.171 | 0.026 (-0.011, 0.064) |
| Marital status | married/living with partner | 6721 (59.59) | ref | ref |
|  | widowed/divorced/separated | 1463 (12.97) | 0.638 | 0.014 (-0.043, 0.071) |
|  | never married | 3095 (27.44) | <0.001 | -0.33 (-0.373, -0.287) |
| Alcohol status | not drinking | 9068 (80.4) | ref | ref |
|  | drinking | 2211 (19.6) | 0.707 | -0.009 (-0.057, 0.038) |

**Table S5** Correlation analysis between blood lipid indicators and other indexes (*n*=541)

|  |  |  |  | LDL | |  | HDL | |  | TG | |  | TC | |
| --- | --- | --- | --- | --- | --- | --- | --- | --- | --- | --- | --- | --- | --- | --- |
|  |  | $\bar{x}\pm S$/ *n*(%) |  | *P* | *b* (95%*CI*) |  | *P* | *b* (95%*CI*) |  | *P* | *b* (95%*CI*) |  | *P* | *b* (95%*CI*) |
| Age (years) | | 32.17 ± 4.397 |  | 0.049 | 0.015 (0.000, 0.030) |  | 0.767 | 0.002 (-0.013, 0.017) |  | 0.215 | 0.012 (-0.007, 0.031) |  | 0.016 | 0.018 (0.003, 0.032) |
| Glucoses (mmol/l) | | 5.096 ± 0.983 |  | 0.000 | 0.138 (0.071, 0.204) |  | 0.000 | -0.184 (-0.249, -0.119) |  | 0.822 | -0.01 (-0.096, 0.076) |  | 0.834 | 0.007 (-0.059, 0.073) |
| BMI (kg/m^2^) | | 21.633 ± 2.851 |  | 0.000 | 0.046 (0.023, 0.069) |  | 0.004 | -0.033 (-0.056, -0.010) |  | 0.000 | 0.070 (0.041, 0.099) |  | 0.007 | 0.031 (0.008, 0.053) |
| Dietary Cu (mg) | | 1.173 ± 0.686 |  | 0.197 | 0.221 (-0.115, 0.556) |  | 0.008 | 0.443 (0.115, 0.772) |  | 0.004 | 0.621 (0.200, 1.042) |  | 0.000 | 0.600 (0.281, 0.918) |
| Dietary cholesterol (mg) | | 466.052 ± 292.229 |  | 0.934 | 0.010 (-0.238, 0.258) |  | 0.000 | 0.477 (0.236, 0.717) |  | 0.043 | 0.322 (0.010, 0.634) |  | 0.000 | 0.415 (0.179, 0.650) |
| Dietary fiber (gm) | | 10.406 ± 6.262 |  | 0.440 | 0.123 (-0.190, 0.436) |  | 0.323 | 0.155 (-0.153, 0.463) |  | 0.246 | 0.233 (-0.162, 0.628) |  | 0.328 | 0.150 (-0.150, 0.45) |
| Dietary zinc (mg) | | 10.194 ± 4.849 |  | 0.105 | 0.311 (-0.065, 0.687) |  | 0.064 | 0.349 (-0.02, 0.718) |  | 0.051 | 0.473 (-0.001, 0.947) |  | 0.008 | 0.484 (0.124, 0.843) |
| Occupation | Unemployed | 206 (38.08) |  | ref | |  | ref | |  | ref | |  | ref | |
| Government employees | | 13 (2.40) |  | 0.892 | -0.030 (-0.472, 0.411) |  | 0.766 | 0.066 (-0.372, 0.504) |  | 0.910 | -0.033 (-0.596, 0.531) |  | 0.573 | 0.122 (-0.303, 0.547) |
| Teachers and healthcare practitioners | | 94 (17.38) |  | 0.359 | -0.090 (-0.282, 0.102) |  | 0.876 | 0.015 (-0.175, 0.206) |  | 0.259 | -0.141 (-0.386, 0.104) |  | 0.034 | -0.201 (-0.386, -0.016) |
| Staff of community and social services | | 129 (23.84) |  | 0.504 | -0.059 (-0.232, 0.114) |  | 0.091 | 0.148 (-0.024, 0.320) |  | 0.587 | -0.061 (-0.282, 0.160) |  | 0.512 | 0.056 (-0.111, 0.223) |
| Business and service employees | | 88 (16.27) |  | 0.023 | -0.228 (-0.425, -0.031) |  | 0.065 | 0.184 (-0.012, 0.379) |  | 0.202 | -0.163 (-0.414, 0.088) |  | 0.317 | -0.096 (-0.286, 0.093) |
| Laborers | | 11 (2.03) |  | 0.419 | -0.197 (-0.675, 0.281) |  | 0.656 | -0.107 (-0.581, 0.367) |  | 0.112 | -0.494 (-1.104, 0.116) |  | 0.051 | -0.458 (-0.918, 0.002) |
| History of diabetes | no | 534 (98.71) |  | ref | |  | ref | |  | ref | |  | ref | |
|  | yes | 7 (1.29) |  | 0.584 | 0.164 (-0.424, 0.753) |  | 0.202 | 0.379 (-0.204, 0.962) |  | 0.138 | 0.566 (-0.182, 1.315) |  | 0.030 | 0.627 (0.060, 1.194) |
| History of  hypertension | no | 540 (99.82) |  | ref | |  | ref | |  | ref | |  | ref | |
|  | yes | 1 (0.18) |  | 0.267 | -0.875 (-2.422, 0.672) |  | 0.002 | 2.41 (0.888, 3.932) |  | 0.831 | -0.214 (-2.186, 1.758) |  | 0.005 | 2.118 (0.631, 3.606) |
| Smoke status | not at all | 526 (97.23) |  | ref | |  | ref | |  | ref | |  | ref | |
|  | yes | 2 (0.37) |  | 0.303 | -0.575 (-1.670, 0.520) |  | 0.457 | 0.412 (-0.675, 1.498) |  | 0.816 | -0.165 (-1.560, 1.230) |  | 0.632 | -0.259 (-1.32, 0.802) |
|  | quit | 13 (2.40) |  | 0.271 | 0.243 (-0.191, 0.677) |  | 0.332 | -0.213 (-0.643, 0.218) |  | 0.182 | 0.376 (-0.177, 0.929) |  | 0.626 | 0.104 (-0.316, 0.525) |
| Drink status | not at all | 495 (91.50) |  | ref | |  | ref | |  | ref | |  | ref | |
|  | yes | 32 (5.91) |  | 0.043 | -0.290 (-0.572, -0.009) |  | 0.594 | 0.076 (-0.204, 0.356) |  | 0.465 | -0.134 (-0.493, 0.226) |  | 0.084 | -0.240 (-0.513, 0.032) |
|  | quit | 14 (2.59) |  | 0.778 | -0.060 (-0.478, 0.358) |  | 0.203 | 0.27 (-0.146, 0.685) |  | 0.461 | -0.200 (-0.734, 0.334) |  | 0.479 | 0.146 (-0.259, 0.551) |
| Moderate PA | no | 317 (58.60) |  | ref | |  | ref | |  | ref | |  | ref | |
|  | yes | 224 (41.40) |  | 0.045 | 0.138 (0.003, 0.272) |  | 0.219 | -0.084 (-0.217, 0.050) |  | 0.644 | -0.04 (-0.212, 0.131) |  | 0.211 | 0.083 (-0.047, 0.214) |
| Vigorous PA | no | 495 (91.50) |  | ref | |  | ref | |  | ref | |  | ref | |
|  | yes | 46 (8.50) |  | 0.070 | 0.220 (-0.018, 0.458) |  | 0.423 | -0.097 (-0.333, 0.140) |  | 0.678 | -0.064 (-0.368, 0.239) |  | 0.194 | 0.153 (-0.078, 0.383) |
| Marital status | unmarried | 7 (1.29) |  | ref | |  | ref | |  | ref | |  | ref | |
|  | married | 534 (98.71) |  | 0.676 | -0.125 (-0.714, 0.463) |  | 0.660 | 0.131 (-0.453, 0.714) |  | 0.280 | 0.412 (-0.337, 1.161) |  | 0.335 | 0.279 (-0.29, 0.848) |


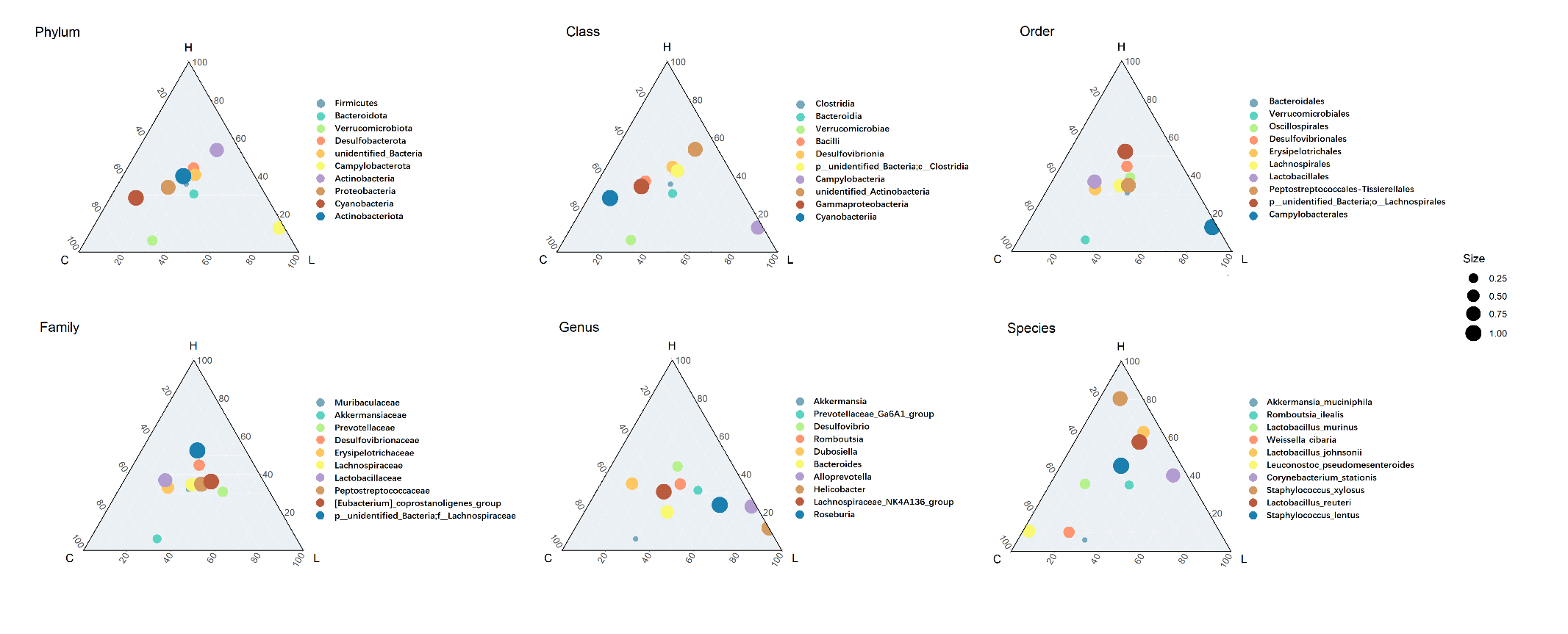


**Fig. S1** The abundance levels of the top 10 gut microbiota in the three groups at different levels (phylum, class, order, family, genus, species) in ternary phase diagrams. C: Control group; L: Low-dose group; H: High-dose group.


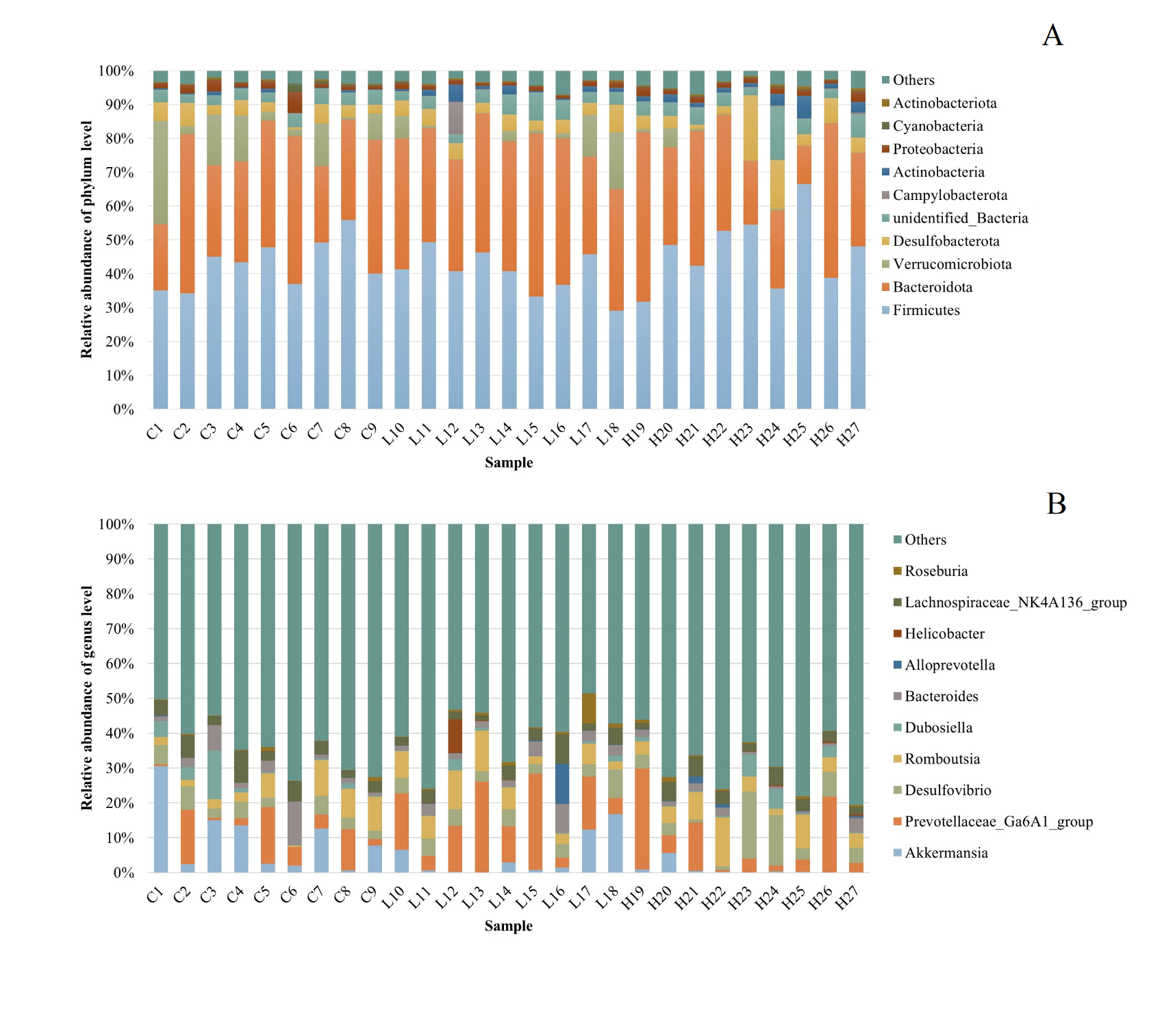


**Fig. S2** Relative abundance of gut microbiota for each animal at the phylum (A) and genus level (B). C: Control group, L: low-dose group, H: high-dose group.
